# Advancing cancer research through 3D cell culture models

**DOI:** 10.17179/excli2025-8563

**Published:** 2025-08-28

**Authors:** Isidora Panez-Toro, Joshua Mountford, Javier Muñoz-Garcia, Dominique Heymann

**Affiliations:** 1 Nantes Université, CNRS, UMR6286, US2B, Biological Sciences and Biotechnologies unit, Nantes 44322, France; 2 Institut de Cancérologie de l’Ouest, Tumor Heterogeneity and Precision Medicine Laboratory, Saint-Herblain 44805, France; 3 University of Sheffield, Medical School, School of Medicine and Population Health, Sheffield, S10 2RX, UK

**Keywords:** 3D cell culture, liquid-based 3D culture, scaffold-based 3D culture, microfluidics, droplet-based 3D culture, organ-on-a-chip

## Abstract

Cancer is a multifactorial disease with cellular proliferative molecular networks and immune evasion properties. The well-known cancer intra- and inter-tumoral heterogeneity presents a notable limitation of the current histological and diagnostic techniques. Thus, biasing the risk of invasiveness and restricting its broader application in oncology in prognostic, survival, and treatment response differences between patients. Monolayer cell cultures have been a consistent *in vitro* model in cancer research throughout time. However, this system fails to replicate the complex pathogenesis of this disease, as key mechanisms underlying initiation, metastasis, drug resistance, and recurrence remain poorly understood. 3D culture models are presented as the most suitable model to better reflect the patient's tumor development. Some methods to introduce the third dimension into cell cultures is by promoting cell-cell interactions to give 3D cell structures, using scaffolds to promote growth beyond monolayers and introducing microfluidic platforms to the system. The present review provides an overview of different techniques to develop 3D culture models in oncology, the advantages compared between monolayer cell cultures, their applications, limitations, and applicability in oncology research.

See also the graphical abstract(Fig. 1).

## Introduction

Cancer is a multifactorial disease that arises from cells adopting abnormal behaviors, enhancing proliferative molecular networks and immune evasion properties. These characteristics lead to immortality and migration/invasion, commonly associated with metastases (Peng et al., 2022[184]; Weeden et al., 2023[240]). One of the most significant challenges researchers face in finding a cure or early diagnosis to improve the prognosis of this disease is the well-known heterogeneity of cancer (Civita et al., 2019[37]; Lunke et al., 2017[143]). Although tissue biopsy pathology has been the standard test for cancer diagnosis, intra-tumoral heterogeneity presents a notable limitation of the technique, biasing the risk of invasiveness and restricting its broader application in oncology. Additionally, the inter-tumor heterogeneity among patients, even within the same cancer type, is characterized by number variability and differences between molecular profiles, which have been correlated with prognostic, survival, and treatment response differences between patients. This concept has gained importance, especially with circulating tumor cell studies. Circulating tumor cells are cells from the primary tumor that are shed into the bloodstream. Analyzing these cells has provided crucial information to determine inter-tumor heterogeneity (Das et al., 2015[43]; Tellez-Gabriel et al., 2019[224]). Considering the intra- and inter-heterogeneity, the histological and molecular analysis of different anatomical areas of different tumor patients gives us a more complete profile of its aggressiveness. Thus, an accurate drug screening is performed to determine the best treatment plan for each patient.

Although monolayer cell culture is widely chosen as an *in vitro* model for cancer research with high reproducibility and low maintenance, life is more complex than a monolayer. In recent years, three-dimensional (3D) cultures have become increasingly significant in oncology research due to their ability to create realistic tumor and microenvironment conditions. Thus, overcoming the limitations of monolayer cultures, particularly regarding their potential to improve the predictability of *in vivo* drug responses, has led to greater drug resistance to various chemotherapeutics than tumor cell cultures grown as monolayers (Abbas et al., 2023[1]; Mohammad Hadi et al., 2020[159]). This underscores the crucial influence of cellular spatial organization and gene expression profiles on overall drug responses (Riedl et al., 2017[199]). In addition, 3D cell culture models allow the capture of metabolic features that play key roles in some cancer types. In ovarian cancer, 3D culture models capture aspects of cholesterol and lipid metabolism, which are both features of metastatic disease (Velletri et al., 2022[235]). In osteosarcoma, 3D culture models mimic the pathophysiology affecting the extrinsic heterogeneity, unlike the solely intrinsic characteristics observed in monolayer cultures. (Pierrevelcin et al., 2022[188]).

These features present 3D models as a promising option for oncology research to study early tumor dynamics. There are still limitations to overcome in extrapolating data to a clinical setting due to the magnitude in size differences with tumors on discovery. In this review, we will discuss different techniques to develop 3D culture models in oncology, aiming to achieve structural complexity, molecular properties, and the heterogeneity of tumor patients alongside discussing their applications and findings in cancer research.

## Cellular Aggregates, Multicellular Tumor Spheroids, Tumorspheres and Tumoroids

Nomenclature of 3D *in vitro* tumor models has not been well standardized, Weiswald et al. (2015[242]) emphasized this in a review which discussed terminology. Four 3D culture models were highlighted: “multicellular tumor spheroids” (3D cultures derived from single cell suspensions), “tumorspheres” (3D models generated from one progenitor cell due to stemness-like properties), “tissue-derived tumor spheres” (3D cultures derived from dissociated patient tumor tissue) and “organotypic multicellular spheroids” (3D cultures derived from non-dissociated patient derived tumor tissue). It has become commonplace for 3D cultures generated from cancer tissues of patients to be referred to as 'organoids' (Drost and Clevers, 2018[51]; LeSavage et al., 2022[132]; Thorel et al., 2024[226]). To avoid confusion between organoids derived from cancerous and healthy tissues, the term tumoroid will henceforth be used to refer to 3D cultures derived from patient tumor tissue.

Another important distinction to consider in 3D cultures is 'cellular aggregation' versus spheroid or tumoroid. Not all cell lines are capable of forming condensed spheroids (Ivascu and Kubbies, 2007[95]), however the introduction of a basement membrane has been shown to improve compact spheroid formation (Dubois et al., 2017[53]; Ivascu and Kubbies, 2007[95]). This may be due to certain cancer cell lines lacking sufficient molecules responsible for cell-cell adhesion, such as E-cadherins, therefore stromal interactions of basement membranes may be necessary to compensate in spheroid formation (Dubois et al., 2017[53]; Han et al., 2021[83]; Mayer et al., 2001[151]; Smyrek et al., 2019[215]). This has been demonstrated *in vitro* with colon cancer, where a P-cadherin deficient line formed less dense spheroids or knockdown of adherens junction proteins yielded looser aggregates (Stadler et al., 2018[218]). It should be considered that looser aggregates may represent metastatic tumor behavior, as isolated “non-spheroid forming” phenotype cells demonstrated greater invasive capacity in colon and ovarian cancer cell lines (Al Habyan et al., 2018[4]; Stadler et al., 2018[218]). Therefore, it is important to consider that the definitions of 3D cultures are not precise, with each model having different characteristics and applications.

**Figure 2:**
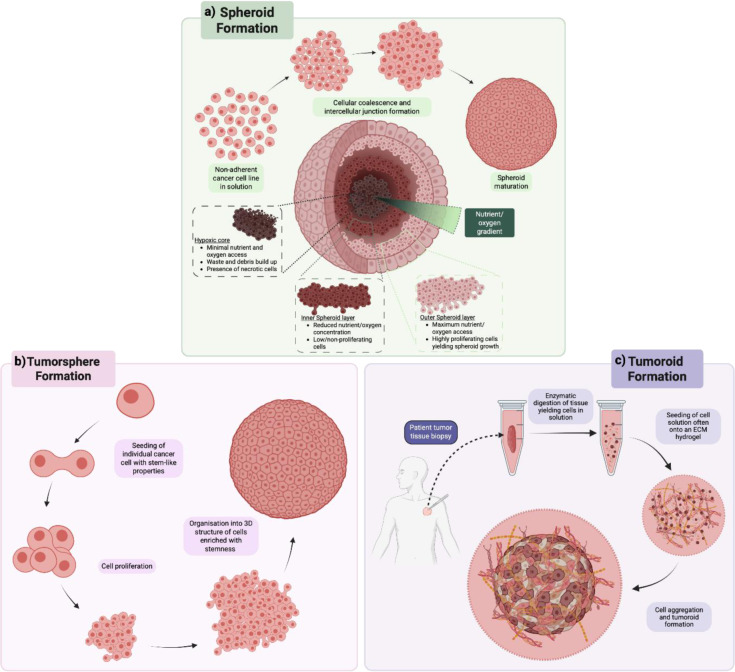
Spheroid, tumorsphere and tumoroid formation. a) Spheroid formation: cancer cells in an ultra-low attachment environment form intercellular connections to form an aggregation of cells that eventually yields a spheroid. At larger spheroid sizes this results in a gradient of nutrients and oxygen throughout the spheroid. The central hypoxic core is limited in access to nutrients and oxygen resulting in necrosis of cells and build up of cellular waste. Beyond this layer with increased nutrient access is a layer of low-proliferating cells and the outer layer is composed of cells with maximum access to nutrients, capable of proliferating. b) Tumorsphere formation: seeding of individual cancer cells with stem-like properties in an ultra-low attachment environment allows proliferation into a tumorsphere formed from one stem-like progenitor. c) Tumoroid formation: Enzymatic digestion and disaggregation of patient tumor tissue yields a solution of cancerous and cancer associated cells. Seeding of this solution into an ultra-low attachment environment or a hydrogel matrix allows the formation of 3D tumoroid structures. Figure created using BioRender.com (https://BioRender.com/tu3quai)

Observations on 3D models of cell-cell interactions *in vitro* dates as far back as 1907 (Wilson, 1907[245]). However the term spheroid was not used until 1971, where V79 lung cells were found to form multicellular multicellular aggregates consisting of an outer dividing layer, dense intermediate layer and a necrotic core (Sutherland et al., 1971[221]). Initial notable key features of this 3D model were improved survivability on radiation treatment when compared to monolayers due to intercellular connections and a threshold size as central cells have limited access to nutrients, hindering growth (Durand and Sutherland, 1972[55]; Folkman and Hochberg, 1973[64]). Lack of vascularization in this model limits nutrient and oxygen accessibility to central cells giving rise to a nutrient gradient resulting in a proliferative outer layer, a slower dividing inner layer and a necrotic core (Folkman and Hochberg, 1973[64]; Zanoni et al., 2020[256]), as demonstrated in Figure 2(Fig. 2).

Solid tumors display intratumoral heterogeneity, whereby the spatial and temporal distribution of a cancer cell within a tumor can give rise to genotypic and phenotypic variations (Dagogo-Jack and Shaw, 2018[41]; Marusyk et al., 2020[148]). Monolayer cultures fail to recapitulate this as cells are incapable of developing cell-cell and cell-extracellular matrix (ECM) and associated signalling pathways (Kim, 2005[108]), and are evenly exposed to metabolites and oxygen (Kapałczyńska et al., 2018[101]; McKeown, 2014[154]). This is evident in the example of tumor oxygen distribution, as uneven oxygen distribution and angiogenesis give rise to tumor hypoxic regions that can fluctuate spatially and temporally (Kumar et al., 2024[118]; Matsumoto et al., 2010[150]). This is of particular importance as hypoxia has been shown to give rise to inter- and intra-tumoral heterogeneity yielding aggressive subclonal populations (Bhandari et al., 2019[21]). *In vitro* 3D tumor models have been shown to develop hypoxic cores in a similar fashion to that of tumors and demonstrated drug resistance often seen in patients (Grimes et al., 2014[75]; Nunes et al., 2019[172]; Pampaloni et al., 2007[178]). Furthermore, Kumano et al. (2024[117]) demonstrated that phenotypic and genotypic effects of hypoxia may irreversibly yield malignant subclones using a tumoroid model. The ability to develop hypoxia indicates how 3D models are better able to represent the *in vivo* environment as they introduce this physiological driver of heterogeneity that monolayers are incapable of.

Additionally, advances in single cell multi-omics are aiding in the identification of subclonal populations in tumors, particularly when studying cells harboring stemness properties, often referred to as cancer stem-like cells (CSCs) (Baysoy et al., 2023[18]; Lawson et al., 2018[126]). Transcriptomic analysis of cells from head and neck squamous cell carcinoma patients identified varying transcriptomic profiles of malignant and non-malignant cell populations. Of interest was a transient partial epithelial to mesenchymal transition (EMT) phenotype was identified, whereby subpopulations of these epithelial derived tumors expressed mesenchymal genes leading to migratory and invasive behavior (Puram et al., 2017[192]). This phenotypic variance is unlikely to be recreated in a monolayer environment, whereas Stadler et al. (2018[218]) isolated distinct “non-spheroid forming” cells on spheroid generation. This subpopulation demonstrated increased migratory capabilities. Furthermore, single cell sequencing techniques have also been applied to 3D cultures to study spheroid heterogeneity and have demonstrated that these models are capable of forming distinct subpopulations in a similar fashion to patient tumors (Muciño-Olmos et al., 2020[165]; Novotný et al., 2020[171]).

Even though spheroids can recapitulate heterogeneity, it should be considered that current cancer spheroid sizes rarely grow beyond 1000 µm in diameter (Boghaert et al., 2017[24]; Ingram et al., 1997[92]; Zanoni et al., 2016[257]). This can be considered a limitation of these 3D models, as this size rarely reflects that of patient tumors. Some such size discrepancies can easily be seen with colon cancer (Saha et al., 2015[206]; Shi et al., 2018[213]), Osteosarcoma (Bieling et al., 1996[22]; Jubelin et al., 2024[98]; Lee et al., 2008[129]) and breast cancer (Narod et al., 2013[169]; Welch et al., 2016[243]; Yakavets et al., 2020[251]), as indicated in Table 1(Tab. 1) (References in Table 1: Bieling et al., 1996[22]; Lee et al., 2008[129]; Narod et al., 2013[169]; Saha et al., 2015[206]; Welch et al., 2016[243]). These experimental spheroid sizes align with Folkman and Hochberg's initial size measurements within 10 days of spheroid generation. Larger sizes could only be achieved beyond 20 days whilst transferring the spheroids to a new flask every 2-3 days (Folkman and Hochberg, 1973[64]).

Despite this shortcoming, spheroids, tumorspheres and tumoroids are still more complex models than monolayers and therefore demonstrate value in modelling pathogenesis of the disease. The following sections will discuss introducing 3D into cell culture techniques alongside their applications and some recent findings advancing cancer research.

## Scaffold-Free 3D Culture Techniques

### Liquid overlay and Hanging drop techniques 

**Figure 3:**
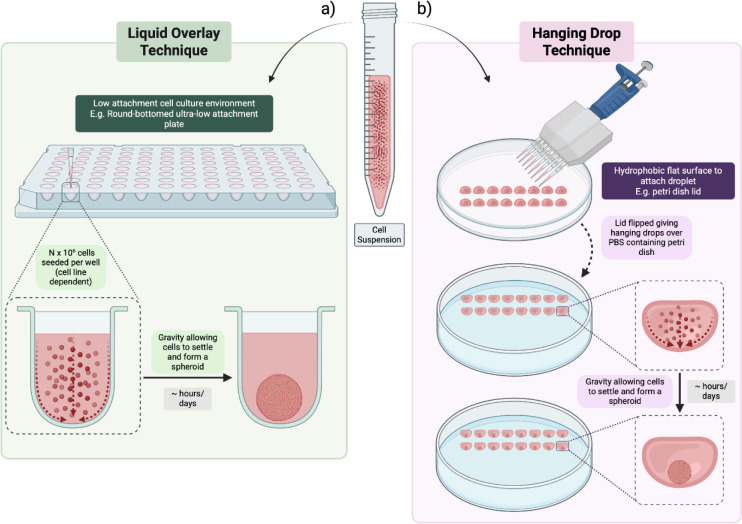
Liquid overlay and hanging drop techniques. a) Liquid overlay technique: seeding cells into a low attachment culture environment (round bottom plate featured here) will cause spheroid(s) to form as adjacent cells form intercellular connections and proliferate as an aggregate. b) Hanging drop technique: seeding cells as droplets onto a hydrophobic surface (culture plate lid here) and then hanging these downward with gravity will cause cells to settle at the bottom of the droplet. Surface tension maintains droplet structure and provides an environment in which cells can form intercellular connections and subsequently a spheroid. Figure created using BioRender.com (https://BioRender.com/rqyhj2e).

Liquid overlay is a simple technique involving culturing cancer cells in standard medium on a non-adhesive surface as illustrated in Figure 3a(Fig. 3). Gravity causes cells to settle at the bottom of the plate where they form intercellular connections and grow to the aforementioned threshold size. Initially this was carried out by treating wells with 0.5 - 1 % agar or agarose gel (Costăchel et al., 1969[40]; Yuhas et al., 1977[255]), but commercially produced ultra-low attachment plates have since been developed to ideally minimize batch variations. However, differences in plate production have been shown to introduce discrepancies in spheroid formation, highlighting potential standardization issues across research from the use of different brands (Xing et al., 2024[249]). Furthermore, the reproducibility of spheroids via the liquid overlay technique is something that has required refining. For example, flat surfaces have been shown to produce multiple unevenly sized spheroids, whereas round-bottom wells tend to promote the formation of one reproducible spheroid (Jubelin et al., 2023[97]; Luan et al., 2022[142]; Tenschert et al., 2022[225]). This is likely due to intercellular connections only being able to form between cells proximal to one another on seeding and the shape of a round-bottom well ensures proximity of all cells. It has been shown that agarose can be manipulated to form a concave meniscus, yielding more uniform spheroids on seeding (Friedrich et al., 2009[68]; Reid et al., 2014[196]), providing an affordable alternative to commercially produced round-bottom plates.

Hanging drop is another simple technique that requires transferring a small volume of cell solution onto a surface, e.g. a plate lid, and allowing the droplet to hang and form a curved surface for cells to gather which can be seen in Figure 3b(Fig. 3) (Foty, 2011[65]; Kelm et al., 2003[105]). The surface tension of the droplet provides an area for cells to settle on, subsequently yielding cellular proximity that promotes intercellular connections akin to the liquid overlay technique. However, a major limitation is that disturbances to this tension may lead to droplet coalescence leading to perturbation of both the droplet and spheroid shape (Blanchette et al., 2009[23]; Zhang and Basaran, 1997[261]). This makes adjusting spheroid media challenging using this technique without transfer to another vessel.

Despite hanging drop seeming quite a rudimentary technique, the groups of Tung et al. (2011[231]) and Kuo et al. (2017[119]) both demonstrated the high-throughput potential of this technique by developing microarray systems that can counter the limitation of droplet coalescence. Kuo's team optimized the ability to generate multiple droplets at once. By using polydimethylsiloxane it was possible to produce droplets as small as 1 µL when supported by collagen fibrils, allowing an increase in spheroids to be produced per surface area. Furthermore, after spheroid growth, droplets of adjacent spheroids, or even growth factors, could be combined with a bridge of media allowing co-culture dynamics or response to a biochemical gradient to be studied. On the other hand, Tung's team developed an array that allowed droplets to be manipulated robotically. This allows the reduction of limitations of consistency and processing speed when compared to manual workflows. Furthermore, this robotic manipulation allowed exchange and adjustment of media, overcoming the limitation of droplet coalescence. They highlighted the feasibility of maintaining spheroids for long-term study by optimizing the hanging drop technique and also demonstrated its usefulness in toxicity testing. 

Meanwhile, other teams have aimed to improve downstream study using these techniques. Zhao et al. (2019[262]) achieved this by combining a 3D printed hanging drop dripper above ultra-low attachment plates. Drop formed spheroids could be released into wells pre-prepared with ECM or chemotherapeutics to allow downstream study of spheroid migration or resistance dynamics respectively. A similar system introducing downstream workflow of hanging drop technique is that of Kim et al. (2022[111]). A chamber containing the drop is instead filled with a hydrogel to break droplet surface tension, meaning release of the spheroid is not necessary, like with Zhao et al's system. Furthermore, they introduced a microfluidic element to the chamber of this model, whereby inlets allow the introduction of cells to facilitate co-culture studies or media additives such as chemotherapeutics to allow the study of resistance in 3D.

Using such co-culture studies also allows introducing complexity to spheroids generated using the liquid overlay and hanging drop techniques. Yakavets et al. (2020[251]) used liquid overlay to generate heterospheroids of MCF7 breast cancer cells and MRC5 fibroblasts to study tumor-stroma interactions. Their research provided a model for fibrosis development in breast cancer patients undergoing radiation treatment, as radiotherapy or addition of transforming growth factor β to spheroids increased collagen production mimicking fibrosis. Similarly, Flörkemeier et al. (2024[63]) also studied tumor-stroma interactions using the liquid overlay technique to generate a co-culture model of ovarian cancer cell lines and fibroblasts. They observed this co-culture system induced changes in spheroid size, morphology and compaction. Interestingly, they also noted that co-cultures heightened spheroid susceptibility to cisplatin, highlighting the importance of including cancer associated cells into 3D systems to potentially capture more representative responses to treatment.

Both hanging drop and liquid overlay techniques have also been useful in evaluating the effectiveness of more novel treatments. Cavaco et al. (2021[30]) used spheroids from breast cancer cell lines to develop a model to test a therapeutic peptide using treatment-resistant cell lines. As is commonly seen with 3D cultures, the IC50s increased by approximately 3-5-fold in all cell lines compared. Meanwhile, Wu et al. (2020[248]) used both techniques for the study of photodynamic therapy kinetics in nasopharyngeal carcinoma spheroids. Not only were evident reductions in apoptosis and necrosis in both spheroid techniques versus monolayers upon treatment observed, but also reduction in drug uptake in both 3D models was noticed. Interestingly, of the examined genes, differential expression between spheroid generation techniques in the LMP1 gene was observed, which is linked to the stabilization of genes of oncogenic potential of the Epstein-Barr virus. This highlights the importance of selection of spheroid generation techniques when carrying out research.

These systems have also been used to study the complexity and heterogeneity of 3D cultures. Dhandapani et al. (2023[47]) used breast cancer spheroids produced by the liquid overlay technique to study the effect of growth in 3D on stemness and resistance to doxorubicin Furthermore, a co-culture of fibroblasts was introduced to study tissue-stroma interactions. As well as the often-reported increase in drug resistance, they noted that the spheroid cultures had upregulated stemness genes Nanog and OCT3/4. Upon fibroblast introduction, increases in genes such as IDO-1, CXCL12 and FAP could also be observed. These genes are found to promote immune cell infiltration but also the induction of an immunosuppressive environment. Close and Johnston (2022[38]) also used the liquid overlay technique to generate spheroids from head and neck squamous cell carcinoma cells lines to develop a novel non-invasive hypoxia probe. The developed HypoxiTRAK™ probe correlates with former hypoxia measurement techniques, but has the advantage that it allows monitoring of hypoxia in real time allowing more high-throughput screening compared to conventional methods. Rodoplu et al. (2022[201]) further built upon the complexity of this technique by combining microfluidic devices with hanging drop techniques. The combined system allowed control of microenvironmental components, treatment application and merging of droplet formed cancer spheroids and/or embryonic organoids. This combinatorial technique allows the study of mechanisms involved in the initiation of angiogenesis.

### Dynamic suspension techniques

**Figure 4:**
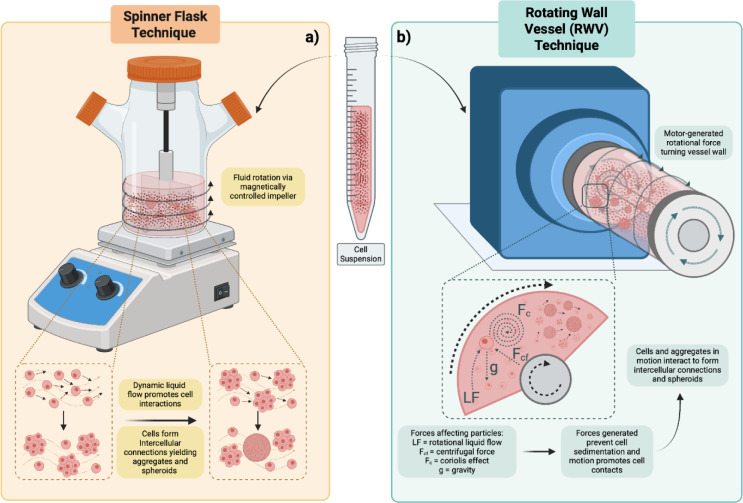
Spinner flask and rotating wall vessel techniques. a) Spinner flask technique: a magnetically driven impeller rotating in the spinner flask ensures that cells are in constant motion and cannot adhere to the vessel surface. The cells in constant motion contact one another resulting in the formation of intercellular connections and subsequent cellular aggregations and spheroids. b) Rotating wall vessel technique: the wall of the vessel rotates at a speed resulting in an environment where cell cultures are suspended in culture without interacting and adhering with the outer vessel wall. The interaction between rotational speed, centrifugal force, the Coriolis effect and gravity maintain cells in motion in the culture medium while minimizing shear stress and turbulence. Cells come into contact with one another, forming intercellular connections to yield aggregates and spheroids. Figure created using BioRender.com (https://BioRender.com/r3867ny)

Dynamic suspension techniques rely on creating a non-static liquid environment in a culture vessel that prevents cells from being able to settle and adhere to surfaces. Continuous collisions of the cells and spheroids maintained in suspension leads to intercellular connections and aggregation, as demonstrated in Figure 4a(Fig. 4) (Frith et al., 2010[69]). As introduced before, Durand and Sutherland pioneered this technique in the early 1970s as they carried out a series of experiments using Chinese hamster V79-171 in spinner flasks using a magnetically driven impeller to generate spheroids which were subsequently used to study the effects of radiation damage in 3D (Durand, 1976[54]; Durand and Sutherland, 1972[55], 1973[56]). In a continuation of this work, Mueller-Klieser and Sutherland (1982[166]) also highlighted the relevance of oxygenation of spheroids developed using these systems as greater oxygenation was observed in dynamic approaches versus static ones. This difference in oxygenation may result in viability discrepancies observed between spheroid generating techniques combined with radiotherapy treatments, as the development of reactive oxygen species will be greater in an oxygenated environment (Perillo et al., 2020[186]). This method has also been used to generate spheroids for implantation *in vivo* (Naus et al., 1992[170]), or as a co-culture method to study pancreatic cancer cell and fibroblast generated ECM interactions to allow study of the tumor microenvironment (Brancato et al., 2017[26]). One consideration of using spinner flasks however is that shear stress provided by such systems run the risk of damaging cells or aggregates and may yield unwanted perturbations in cell metabolism (Henzler, 2000[85]; Mollet et al., 2004[161]).

A system that has been found to overcome this is the rotating wall vessel (RWV). During the late 1970s and early 1980s the National Aeronautics and Space Agency (NASA) demonstrated that red blood cells in vitro will aggregate in space under zero gravity (Dintenfass, 1986[49]). This research paved the way in the early 1990s for a team at NASA to generate a method that minimizes the shear stress produced by conventional bioreactors (Schwarz et al., 1992[211]). Observations from plant studies identified that centrifugal force from clinostats can remove gravity as a stimulus to plants (Dedolph and Dipert, 1971[45]). This inspired the production of the RWV, as continuous rotation of the vessel results in the contained medium matching the speed and direction of the wall, causing cells, often attached to microcarriers, to experience what is close to 'simulated microgravity' with minimal shear force, as detailed in Figure 4b(Fig. 4) (Hammond and Hammond, 2001[82]; Schwarz et al., 1992[211]; Unsworth and Lelkes, 1998[232]). Furthermore, the silicone membrane composition of this device allowed oxygen diffusion into the culture, aiding growth. A team at the Huntington medical research institutes used such a bioreactor to successfully culture spheroids of 14 cancer cell lines, a healthy prostate fibroblast line and a co-culture of prostate cancer with the aforementioned fibroblast line without the use of microcarrier beads. In doing so they highlighted how spheroids generated via this technique were more than simple aggregations of cells, as when compared to pellets of monolayer cultures they exhibited distinct tissue-like heterogeneity (Ingram et al., 1997[92]).

McNeill et al. (2018[155]) demonstrated the effectiveness of the RWV method by using ECM coated microcarriers co-cultured with mesenchymal stem cells (MSCs) and osteosarcoma cells to study bone-tumor interactions. They identified that Wnt inhibition by the osteosarcoma cell lines could inhibit osteogenic differentiation in MSCs. Alongside this, it was also observed that the osteosarcoma cells demonstrated invasive behavior and could displace the MSCs from the microcarriers. Using simulated microgravity however comes with considerations as reducing shear stress by limiting gravity as a variable introduces a variable in and of itself. Growing cells under these conditions has been shown to have an impact on cell cycle progression (Moos et al., 1994[164]; Vassy et al., 2003[234]), cell death (Arun et al., 2017[11]; Pisanu et al., 2014[191]), and cell migration (Ma et al., 2014[145]). Furthermore, a tumor growing inside of a person will not be experiencing microgravity. Even when taking into consideration non-anchored circulating tumor cells (CTCs), these cells still experience high levels of shear stress (Lin et al., 2021[138]), unlike those in the RWV. Whilst this technique is a valid form of producing spheroids, these limitations need to be considered in terms of extrapolating findings to pathological conditions.

The main advantage of these dynamic suspension techniques is that they have been shown to be an effective method to maintain large batches of spheroids over extended periods of time (Chen et al., 2019[31]; La Rocca et al., 2023[122]; Massai et al., 2016[149]), with viable growth of endothelial cells for up to one month even being reported (Franchi-Mendes et al., 2021[66]). This advantage is evident when compared to hanging drop and liquid overlay techniques, as previously mentioned these methods often result in the production of one spheroid per well, making scale-up much more cumbersome. Liu et al. (2022[140]) however took advantage of both techniques by initially generating spheroids in a chip containing ultra-low attachment microwells via the liquid overlay technique. These spheroids could then be transferred to a spinner flask, where both media input and spinning speed could be adapted to control spheroid size. They were successfully capable of generating up to 841 spheroids at a time highlighting the high-throughput potential that can be achieved with these 3D techniques. However, the scalable nature of dynamic techniques also acts as a limitation as it is not possible to follow individual spheroids throughout this process and isolating singular spheroids proves to be a challenge (Ingram et al., 1997[92]; Rodday et al., 2011[200]). This therefore means that while dynamic techniques may be useful in generating large quantities of spheroids and it may possible to monitor the effects of therapeutics on a variable such as average spheroid size at the experimental end-point, it is not as easy to follow the dynamics of the treatment over time on the spheroid itself.

### Magnetic levitation techniques

**Figure 5:**
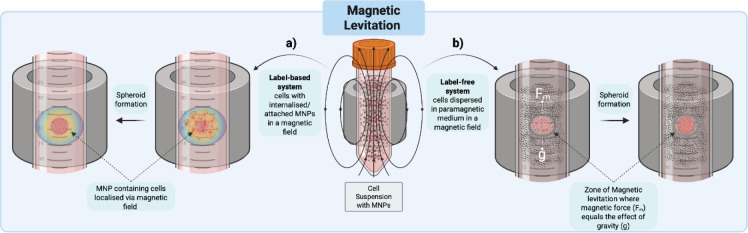
Magnetic levitation. a) Magnetic nanoparticle (MNP) label-based system: Cells are labeled with MNPs, via uptake or attachment to the cell surface, allowing the manipulation of cell location using a magnetic field. In this example a round magnet maintains cells in a central area of a culture suspension where intercellular connections can form allowing spheroid formation. b) MNP label-free system: Cells can be placed in a paramagnetic medium where MNP uptake does not take place, the combination of the magnetic field and paramagnetic medium creates an upward force that counteracts that of gravity on the cells. In this example a round magnet creates a zone of magnetic levitation, localizing cells resulting in the formation of intercellular connections and a spheroid. Figure created using BioRender.com (https://BioRender.com/y3tr263)

Originally magnetic nanoparticles (MNPs) had been developed with their therapeutic potential in mind, with uses such as magnetic hyperthermia therapy or as contrast agents to aid imaging (Dobson, 2008[50]; Pankhurst et al., 2009[179]). However, in the field of tissue engineering it was demonstrated that attaching cells to MNPs allowed cell patterning via spatiotemporal adjustment on a flat surface using magnetism, with this being achieved in 3D to create tissue tubules (Ino et al., 2007[93]; Ito et al., 2005[94]). Building on this approach, Souza et al. (2010[217]) showed that glioblastoma cells that had taken up MNPs could be levitated using a magnetic field. This magnetic field could then be used to manipulate levitating cells into proximity with one another to induce intercellular connections, yielding a spheroid. This is demonstrated in Figure 5a(Fig. 5). This led to the development of an experimental method allowing easy spheroid generation, manipulation and transfer by taking advantage of magnetism (Haisler et al., 2013[81]). Perez et al. (2020[185]) demonstrated how this method could be used to reproducibly generate larger glioblastoma cell line spheroids when compared to the hanging drop method. Uniquely, it was shown that this method even allows spheroid surface tension measurements, allowing spheroid cohesiveness to be monitored. It was suggested that perturbations in this cohesiveness value could yield potential clues into migration and invasion.

Kim et al. (2013[107]) adapted the magnetic levitation system by altering magnet shape to adjust the magnetic field. They found using a pin shaped magnet above the cell culture would aid in focusing cells at a specific location which could increase cellular proximity and more efficiently yield intercellular connections. Timm et al. (2013[227]) also similarly demonstrated the potential behind adjusting the magnetic field by designing a ring-shaped magnet that could yield a ring-shaped spheroid. This model allows the study of cell migration in 3D as the rate at which the center of the ring fills can be monitored to give a quantifiable readout of migration. Whilst cancer cell lines weren't used here, the ability to observe migration in 3D is highly relevant as it could be used to study tumor cell migration and invasion and may reflect the metastatic potential of the cancer modelled. Furthermore, the accessibility and high throughput nature of this model may allow the study of therapeutics to treat invasion and metastases in both spheroid and tumoroid models.

Jaganathan et al. (2014[96]) demonstrated the effectiveness of magnetic levitation in co-culture spheroid generation by using stromal cells alongside a breast cancer cell line. This model was found to more accurately replicate drug response compared to monolayer culture models, as the increased ECM production better reflected the *in vivo* effect of stroma inhibition of drug penetration. Furthermore, this methodology also shows promise in generating tumoroids. Ferreira et al. (2019[60]) developed a 'salisphere' organoid using magnetic levitation of porcine salivary gland derived cells that responded to external stimulation to produce salivary enzymes. Whilst this model is not cancer-based, this team hopes that it will aid in the creation of transplantable tissues that can be used to treat head and neck cancer patients that are experiencing hypofunction of salivary glands due to radiotherapy.

Using magnetic levitation offers the potential for increased manipulation of cells in 3D, but this method is still a double-edged sword in that non-native MNPs are required for these manipulations. Superparamagnetic iron oxides (SPIONs) or gold nanoparticles are often used in such methodology (Kappes et al., 2022[102]; Kim et al., 2013[107]), for example the biocompatible gold and iron oxide Nanoshuttle™-PL has shown frequent use (Fernandez-Vega et al., 2022[59]; Hou et al., 2018[88]; Leonard and Godin, 2016[131]; Timm et al., 2013[227]). Despite biocompatibility of such MNPs at certain concentrations and in various cell lines, gold and iron nanoparticles have demonstrated cytotoxicity (Lewinski et al., 2008[134]; Malhotra et al., 2020[146]). For example, Du et al. (2017[52]) found that SPIONs induced reactive oxygen species formation in Osteosarcoma cells, leading to apoptosis. Furthermore, both gold and iron nanoparticles have been shown to affect cell morphology (Buyukhatipoglu and Clyne, 2011[27]; Patra et al., 2007[183]), possibly due to cytoskeletal perturbations (Ali et al., 2017[6]; Choudhury et al., 2013[36]; Gupta and Gupta, 2005[80]; Královec et al., 2020[116]). To try and counter such negative effects, the MNP coating can be adjusted to limit variables such as aggregation to improve (Schubert and Chanana, 2018[210]; Takahashi et al., 2006[223]). Therefore, it is necessary to optimize nanoparticles for magnetic levitation as cellular uptake is essential for this method to function, but effects on viability and morphology need to be taken into consideration for effective spheroid formation.

An alternative approach to circumvent issues of toxicity is to instead use n[175]egative magnetophoresis, which entails utilizing a paramagnetic medium which can be manipulated around the cells as opposed to labelling with MNPs as highlighted in Figure 5b(Fig. 5) (Anil-Inevi and Ozcivici, 2025[8]; Zhang et al., 2018[259]). Parfenov et al. (2020[180]) demonstrated this with pre-formed chondrosarcoma spheroids, where a gadolinium (III) chelate containing medium under a strong magnetic field created a 'zone of stable levitation' in which spheroids could be maintained. This technique was particularly of interest as the concentration used was 100 times less than what is usually reported as toxic. The utility of this technique was highlighted by Anil-Inevi et al. (2021[7]), as they demonstrated the ability to manipulate and merge spheroids. Furthermore, various teams have also demonstrated the ability of negative magnetophoresis to generate 3D cellular structures. Magnetic field manipulation has shown the potential to generate tube-like 3D aggregations of cells (Anil-Inevi et al., 2018[9]), rounded spheroids (Moncal et al., 2022[163]; Tocchio et al., 2018[229]); or irregularly shaped spheroids (Onbas and Arslan Yildiz, 2021[175]).

## Scaffold-Based 3D Culture Techniques

In the aforementioned techniques gravity is often a key variable in bringing cells into proximity to form intercellular connections, yielding 3D structures. However, the third dimension can be introduced into a cell culture system by using scaffolds composed of either a natural or synthetic ECM (Kyburz and Anseth, 2015[121]). Scaffolds cell growth in the Z-axis beyond the monolayer via cell-ECM connections, as opposed to inducing 3D arrangement of cells via cell-cell connections as seen with spheroid generation techniques.

Endogenous ECM produced by cells is composed mainly of proteoglycans and fibrous proteins. It is a dynamic scaffold that functions by inducing biochemical and biomechanical effects on adjacent cells (Frantz et al., 2010[67]). Integrins allow individual cells to sense and interact with the varying components within the ECM. Furthermore, the physical properties of the ECM, such as stiffness, influence cells mechanically and trigger various signal transduction pathways (Kechagia et al., 2019[103]). It is therefore no surprise that cancer cells have unique interactions with the ECM, as tumors produce both ECM components and enzymes involved in its remodeling (Winkler et al., 2020[247]). It should also be considered that different tumors will experience vastly different ECM properties based on their unique tumor microenvironment (TME). For example, an osteosarcoma may manipulate osteoblastic and osteoclastic cells in the local TME to remodel bone ECM, potentially resulting in osteoid formation (Grünewald et al., 2020[77]; Beird et al., 2022[19]). Meanwhile, lung cancers can induce collagen crosslinking via lysyl-oxidases altering the stiffness of the surrounding tissue (Parker and Cox, 2020[181]). Whilst in both examples ECM stiffness and composition are manipulated, these two TMEs will be unique in terms of composition and mechanics. Therefore, scaffold models for specific cancers and their TME will need to be tailored to better reflect the *in vivo* pathology.

Furthermore, tumors can recruit and reprogram stromal cells to aid in TME remodeling. This is most commonly seen with cancer associated fibroblasts (CAFs) which can secrete ECM components, alongside supplying metabolites, aiding tumor pathogenesis (Avagliano et al., 2018[13]; Santi et al., 2018[207]). The complex interplay between cancer cells, stromal cells and the architecture of the stroma is not something that can be achieved in monolayers. Therefore 3D models are necessary to mimic these interactions to better understand the role of the ECM in processes such as metastasis, immune evasion or tumor growth (Closset et al., 2023[39]; Winkler et al., 2020[247]).

It wasn't until the 1970s where Molecular biology-led research in the 1970s into cell-ECM interactions started to truly tease at the function and composition of the ECM (Kefalides, 1973[104]; Piez, 1997[189]). Various teams were beginning to characterize the components of a suspected cartilage-based ECM produced by a chondrosarcoma maintained in rats (Choi et al., 1971[35]; Oegema et al., 1975[173]; Smith et al., 1975[214]). Concurrently, Orkin et al. (1977[176]) were researching a murine chondrosarcoma, where they identified that this model produced excess amounts of a basement membrane as opposed to the expected cartilage-based ECM. This mouse chondrosarcoma was subsequently identified as Engelbreth-Holm-Swarm (EHS) sarcoma. The excess basement membrane produced by this sarcoma laid the foundation for research into the compositional components of the ECM in the 1980s (Kleinman et al., 1982[113]; Timpl et al., 1979[228]), and lead to the development of Matrigel^TM^, arguably one of the most widely used ECM mimics in academic research today (Kleinman and Martin, 2005[113]). Maniotis et al. (1999[147]) demonstrated the importance of Matrigel^TM^ l as a scaffold in a study on melanoma cell line vasculogenesis. The 3D model induced a more pluripotent phenotype in putative aggressive melanoma cell lines. Furthermore, it was identified that vascular channels were created in Matrigel^TM^ despite the absence of blood vessels, unveiling a potential mechanism for angiogenesis independent of endothelial cells. This influential study highlighted the potential of Matrigel^TM^ to study unique processes in tumor development. Beyond this, cancer research using Matrigel^TM^ has varied from a platform to study migration and invasion (Aslan et al., 2021[12]; Pijuan et al., 2019[190]), to aiding in spheroid formation (Badea et al., 2019[14]; Gopal et al., 2021[72]), or to study the effects of tumor-ECM interactions on cell phenotype (Li et al., 2023[135]; Weaver et al., 1997[239]).

**Figure 6:**
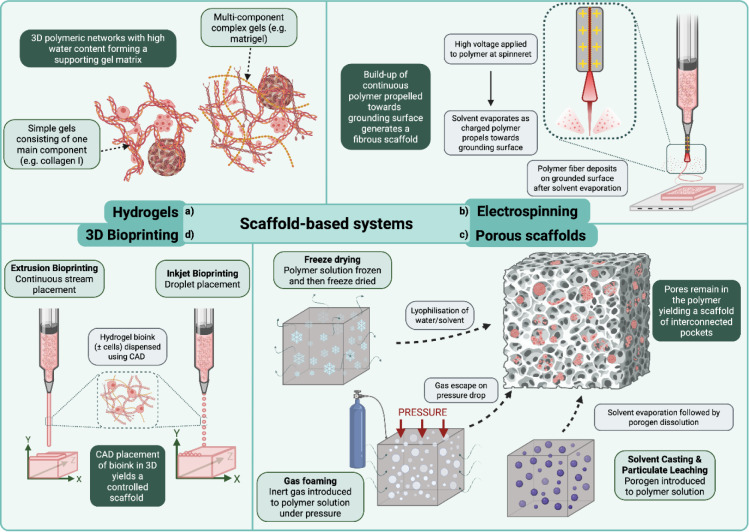
Scaffold-based systems. a) Hydrogels: are polymeric networks with high water content, forming a gel-like material that can suspend cells and support 3D cultures. They can be composed of one or more polymers to give varying complexity. b) Electrospinning: a solution of charged polymer is propelled towards a grounding surface. Solvent evaporation during this process yields a continuous fiber which can be overlaid to generate a 3D fibrous scaffold that can support cell growth and facilitate directional migration. c) Porous scaffolds: a polymer solution is processed via one of several methods: freeze drying, gas foaming or solvent casting and particulate leaching. These processes generate pores within the polymer, which create interconnected pockets that support cell growth and enable migration into the scaffold d) 3D Bioprinting: A bioink consisting of a polymer, with or without cells, is placed in a controlled manner in 3D using a bioprinter combined computer aided design (CAD). Bioink placement allows the creation of spatially controlled scaffolds, either preloaded with cells or designed for subsequent cell seeding. Figure created using BioRender.com (https://BioRender.com/us6bccq)

A major shortcoming to be considered with Matrigel^TM^ is its poorly defined and complex composition, with an estimated 1851 unique proteins identified across multiple batches (Hughes et al., 2010[90]; Kozlowski et al., 2021[115]). It has been shown that the presence of growth factors such as transforming growth factor β (TGFβ) or epidermal growth factor (EGF) likely affects cellular behaviors, potentially introducing extraneous variables into experiments (Vukicevic et al., 1992[237]). For example, it may be difficult to determine if cellular response is due to membrane stiffness or differing levels of growth factors in an experiment using varying concentrations of Matrigel^TM^. Growth factor reduced (GFR) Matrigel^TM^ has been proposed as an alternative to control for this variable. However, batch-to-batch similarity of protein composition of GFR Matrigel^TM^ has been reported to potentially be as low as 53 % when supplied by different manufacturers (Hughes et al., 2010[90]). The confounding variables of undefined composition and batch variation risk may introduce discrepancies in results and hinder standardization due to Matrigel^TM^ complexity (Aisenbrey and Murphy, 2020[3]). As of 21/03/2025 a search of “matrigel NOT review[pt]” using Pubmed (https://pubmed.ncbi.nlm.nih.gov/) yields 14,186 publication results. Interestingly, articles for this search on this platform appeared to peak in 2013 with 794 results and a downward trend has been seen since with approximately half this amount in 2024. This downward trend in Matrigel^TM^ use may be due to the aforementioned shortcomings and continuing development of alternative hydrogels and scaffold-based systems. The following section will highlight various scaffolds and technologies used to generate 3D cell culture models capable of mimicking the TME. These models aim to better reflect cancer cell behavior than that of monolayer cultures, aiding in the study of cellular mechanisms and the development of therapeutics. Figure 6(Fig. 6) demonstrates the scaffolds or production methods covered in the following section.

### Hydrogels

Hydrogels have garnered significant interest since Wichterle and Lím (1960[244]) pioneered a synthetic biocompatible hydrogel and the aforementioned discovery of EHS produced Matrigel^TM^. Hydrogels are scaffolds consisting of cross-linked polymers capable of absorbing large concentrations of water, where composition can be altered to mechanically affect cells in terms of stiffness or promote cell adhesion via interaction with integrins (Caliari and Burdick, 2016[29]). Fischbach et al. (2009[62]) highlighted the importance of cell-ECM interactions in oral squamous cell carcinoma and breast cancer cell lines. They developed a synthetic alginate hydrogel modified with arginyl-glycl-aspartic acid (RGD) domains that allow integrin binding. Although the alginate hydrogel alone did not support cell adhesion, this condition still saw an increase in the pro-angiogenic secreted factor interleukin-18 (IL18) by 17-fold compared to monolayers. However, introducing the RGD domain to this hydrogel further increased IL18 secretion to 35 times that of monolayer cultures. These results not only highlighted the importance of working in 3D, but also demonstrated the importance of introducing integrin binding into tumor models to study important tumorigenic processes such as angiogenesis.

In another example, Below et al. (2022[20]) developed a synthetic hydrogel composed of polyethylene glyclol (PEG) to mimic the ECM of the pancreas to study pancreatic cancer tumoroid development. Using data from *in vivo* pancreatic cancers they generated a 'matrisome' of matrix binding proteins, which allowed the identification of essential ECM components such as certain laminins, fibronectins and collagens necessary to mimic cell-ECM adhesion *in vitro*. Moreover,* in vivo *pancreatic cancer tissue stiffness was used to determine a physiological relevant stiffness for study. Fine-tuning these characteristics allowed the development of a hydrogel model optimized for pancreatic tumoroid growth with customizable stiffness to measure its impact on tumor growth. Alongside this, co-cultures were carried out using this hydrogel with stromal cells. Not only were cell-specific signaling molecules, such as CXCL12, found to be upregulated, but stromal cells also integrated into the pancreatic cancer tumoroids. This study demonstrated how layers of complexity can be introduced into a synthetic scaffold to more closely replicate the patient TME. On the other hand, instead of using this bottom-up approach of developing a representative hydrogel, Kim et al. (2022[109]) used a decellularization process of gastrointestinal tissue to generate an ECM hydrogel that can support healthy cell organoids or colon cancer tumoroids. They demonstrated this scaffold could support tumoroid formation, whilst addressing batch-to-batch variability of Matrigel^TM^ through sample pooling, and overcoming the limitation of synthetic scaffolds being unable to fully replicate the patient TME. These papers demonstrate how the natural and synthetic dichotomy are a balancing act between complexity and control. Synthetic scaffolds allow customization to be introduced to the system to allow study of the effects of individual components, whilst natural scaffolds offer the ability to study a more faithful representation of the TME.

Further complexity can be introduced by combining scaffolds to give rise to a more heterogeneous environment. Pal et al. (2019[177]) achieved this by embedding either breast or gastric cancer cells into a gelatin methacrylamide (GelMA) hydrogel which was then loaded onto a fibrous Poly Lactic-co-Glycolic Acid (PLGA) scaffold. The aim of this work was to study migration mechanisms of cancer cells interacting with environments of variable stiffness in a way that replicates heightened stiffness often seen with solid tumors. Alongside an increase in cancer cell proliferation, the combined scaffold gave rise to a subpopulation of cells with unregulated expression of several genes involved in EMT. Not only did EMT markers such as N-cadherin, vimentin and Fibronectin increase, but also EMT-associated transcription factors such as Zeb1, Twist2 and Snail1 saw upregulation. Furthermore, upregulation of the stemness marker CD44 was observed in the breast cancer line MDA-MB-231 on the combined scaffold. This work highlighted how using a singular scaffold is still reductionistic when studying cancer, as tumors will experience varying and dynamic TMEs. Furthermore, the extra layer of complexity from the combined scaffolds gave clues on stemness phenotypes which are often associated with metastasis, resistance and recurrence (Yang et al., 2020[252]), potentially providing a model to study treatments for cells demonstrating these phenotypes.

### Electrospinning of fibrous scaffolds

Electrospinning is a common method for the production of fibrous scaffolds. A polymer solution is passed through a metallic nozzle (spinneret) whilst experiencing a high electrical voltage, applying an electrical charge to the expelled droplet. The polymer is then attracted to a surface of ground or opposite charge and propelled from the droplet in the form of a jet, during which the solvent evaporates to yield a continuous polymer fiber that can be overlayed to form a scaffold (Li et al., 2021[137]). Variables such as distance to collecting surface (Hekmati et al., 2013[84]), polymer and solvent solution (Shen et al., 2022[212]), polymer composition gradient (Grey et al., 2013[74]), number of spinnerets (e.g. dual extrusion electrospinning) (Levorson et al., 2013[133]), or post-fabrication modifications (e.g. addition of functional groups) (Mohammadalizadeh et al., 2022[160]), can all be adjusted to give rise to fibers as thin as nanometers with differing structural and biochemical properties (Gonçalves et al., 2021[71]; Rahmati et al., 2021[194]; Zhou et al., 2018[264]). Furthermore, electrospun fibers can be embellished upon to introduce extra complexity to the system. For example, porosity can be introduced to electrospun fibers by using techniques such as gas foaming with CO2 or nanofibers produced can be used as a component in hydrogels for use in the creation of bioinks for 3D-printing (Jun et al., 2018[99]; Kamaraj et al., 2024[100]). The overlaying of fibers produces a 3D scaffold with interfibrillar spaces which cancer cells can migrate and invade into, akin to tissue invasion in patients (Xue et al., 2018[250]).

Saha et al. (2012[205]) took advantage of the ability to control fiber orientation during electrospinning to study the effect of directionality using poly(ε-caprolactone) (PCL). This scaffold aimed to mimic parallel fiber alignment in the ECM that has been observed in primary breast carcinoma. Seeding of breast cancer cells on aligned fibers resulted in greater cell elongation coupled with upregulation of genes associated with metastatic EMT, such as cytokeratin 14 (CK14) or smooth muscle actin (SMA). Li et al. (2024[136]) also used aligned electrospun PCL to tease apart potential mechanisms behind breast cancer cell migration. Again, the aligned orientation gave rise to more unidirectional elongated spindle-like cells, however they also observed greater motility on aligned scaffolds. This directionally-induced increase in motility correlated with increased levels of caveolin-1 (CAV1). CAV1 was shown to induce F-actin polymerization which in turn gave rise to focal adhesion complex formation. Moreover, it was determined that CAV1 levels correlated with increased translocation to the nucleus of Yes-Associated Protein (YAP), a transcription factor implicated in mechanotransduction. This importance of CAV1 in invasion was further supported *in vivo* as CAV1 silenced cells showed reduced invasion into surrounding murine muscle and fat tissue. These models indicated that even subtle features of the ECM, such as fiber direction, can affect complex signaling pathways that can impact severe cancer outcomes, such as risk of metastasis.

Guiro et al. (2015[79]) also used electrospinning of a PCL scaffold with the aim of studying the effect of scaffold alignment on migration. They generated a carboplatin resistant breast cancer cell line that demonstrated a dormant phenotype with upregulation of stemness genes such as OCT4 and BCL2 when compared with the parental line. Both lines demonstrated the ability to invade into interfibrillar spaces of the randomly aligned scaffold and to directionally align with fibers, indicating the suitability of this 3D model to study migration as a model of invasion and metastasis. However, an interesting observation was that the resistant line did not appear to change phenotype upon seeding onto the scaffold, maintaining the dormant phenotype observed in monolayer. This is in contrast to the parental line, which adopted a more dormant phenotype on scaffold seeding in a similar fashion to the resistant line. Not only did this data highlight the importance of adjusting variables such as ECM alignment to observe phenotypic changes, but also hints at the potential for scaffolds to be used in drug resistance studies. 

An electrospun PCL scaffold has also been used to study the interplay of stromal cells and ECM in the TME by Balachander et al. (2018[15]), as this system was combined with patient derived cancer associated fibroblasts (CAFs). CAFs seeded onto the scaffold were perceived to be in a more activated state due to increased levels of αSMA when compared to monolayer cultures. The scaffold-associated CAFs also demonstrated upregulation of pro-inflammatory genes of the NF-κβ pathway, often associated with increased tumor invasiveness. These experiments indicated the importance of studying the effects of 3D cell culture on cancer associated cells as well as cancer cells themselves. As this activated phenotype is more likely to reflect CAFs of aggressive tumors in patients. Furthermore, MDA-MB-231 breast cancer cells co-cultured with these activated CAFs saw significant increases in the stemness associated transcription factors BMI1 and NANOG. A frequent emerging pattern is the role of a stemness phenotype in more aggressive cancers, this model highlights how moving beyond monoculture monolayer studies is necessary to replicate this phenotype to yield a platform with which to study and treat stemness.

The ability to increase complexity of scaffold composition produced by electrospinning has also shown advantages in terms of inducing cell-cell contacts as well. Girard et al. (2013[70]) introduced PEG as a co-polymer to PLGA and combined this with polylactic acid (PLA) to form a '3P' scaffold. Small spheroids that resemble micro metastases formed on these scaffolds. These spheroids showed increased levels of the mesenchymal marker vimentin and decreased levels of E-cadherin when compared to cells on PLGA scaffolds or in monolayer. Phosphoinositide 3-kinase (PI3K) and Mitogen Associated Protein Kinase (MAPK) inhibitors were found to prevent this spheroid formation and size in a dose-dependent manner. These are central to the epithelial-to-mesenchymal (EMT) process, implying that spheroid formation on such scaffolds may be correlated with a shift towards the mesenchymal phenotype. This scaffold was also found to successfully promote tumoroid formation on seeding of singular cells of tumor biopsies, which also experienced inhibited growth on addition of PI3K and MAPK inhibitors. However, tumoroids demonstrated greater resistance to these inhibitors when compared to the spheroids. These findings again indicate the importance of scaffold interactions and the ECM in influencing cell phenotype. Moreover, it was highlighted that even though spheroids formed using such a scaffold may better reflect patient tumors than monolayers, they are still limited when compared to that of a patient-derived tumoroid model.

### Porous scaffold generation

Whilst electrospinning generates fibers with interfibrillar spaces to facilitate growth and invasion as an ECM mimic, another approach is to develop a scaffold containing pores with which cells can be seeded and to invade. Formation of these scaffolds is usually achieved by generating air pockets in a polymer via freeze drying, foaming or Solvent Casting and Particulate Leaching (SCPL) (Loh and Choong, 2013[141]; Mikos and Temenoff, 2000[156]). Freeze drying, or lyophilization, requires freezing of a polymer hydrogel to yield ice crystal nucleation. The freeze-drying process then removes water from the system via sublimation, resulting in pores left at the sites of nucleation supported by the surrounding polymeric structure (Grenier et al., 2019[73]). Gas foaming entails introducing an inert gas to a hydrogel polymer at high pressure. A subsequent drop in pressure then results in a state of gas supersaturation, followed by bubble formation due to nucleation of the gas. This results in pores being produced in the shape of air bubbles as the gas leaves the system (Dehghani and Annabi, 2011[46]). Meanwhile, SCPL instead entails mixing the polymer scaffold solution with an insoluble salt porogen, an example being NaCl, and introducing this mix to a cast. Thermally induced solvent evaporation leaves behind a polymer-porogen complex, after which a porogen specific solvent can be introduced to the system, water in the case of NaCl, yielding a porous scaffold (Sola et al., 2019[216]).

Yang et al. (2015[253]) demonstrated how a gelatin foam could be used to generate glioblastoma tumoroids from within the porous structure. Stem-like cells cultured in the gelatin scaffold maintained stemness gene regulation, such as OCT4, comparably to spheroids grown in low attachment conditions, whilst in monolayers such genes were found to be heavily downregulated. Furthermore, cells grown in the scaffold demonstrated spheroid-comparable chemoresistance against therapeutics such as Irinotecan and Fluorouracil. However, the hypoxia-activated chemotherapeutic tirapazamine was found to be more cytotoxic in the 3D models when compared to monolayers, this coincided with an upregulation of hypoxia-inducible factor-1α (HIF-1α). This finding is not surprising considering it is consistent with the aforementioned findings that a hypoxic core is common in 3D cell cultures, whereas hypoxia is not observed in standard monolayer cultures. This study demonstrated how porous scaffolds can be used as valuable models to study the stemness phenotype in cancers and gives clues as to how residual populations after treatment can give rise to recurrence due to increased drug resistance. Furthermore, developing tumoroids on this scaffold demonstrated potential in personalized medicine, as tumoroids generated from patient cells may be more representative of therapeutic response in the clinic. Zhou et al. (2023[265]) also studied drug response in glioblastoma cells, however they highlighted the high-throughput potential of a porous scaffold. They demonstrated that a 96-well array containing a chitosan-hyaluronic polymer could be subjected to freeze drying to yield a plate of porous scaffolds, after which cells could be seeded and treated with therapeutics. Cells seeded on the scaffold upregulated genes associated with drug resistance, such as CD44, ABCG2 and MGMT in a manner correlating with pore size and cell line. Compared to monolayer cultures, all cell lines demonstrated greater resistance to temozolomide at all pore sizes of the scaffold. This study demonstrated the ability to scale up freeze-dried scaffolds to take advantage of the drug resistance properties observed in 3D cultures to act as a better representation of *in vivo* and clinical responses.

Porous scaffolds have also been used to study mechanical variables on tumor development. Le et al. (2021[127]) developed a natural chitosan-alginate scaffold using freeze drying to study the effect of tissue stiffness on breast cancer tumorigenesis. Since this polymer combination lacks sites to form cell-ECM focal adhesions, it allowed this team to control the singular variable of stiffness by adjusting polymer concentration. Scaffold seeding caused a shift from the standard spindle shape to aggregates of a circular morphology in the MDA-MB-231 cell line and increasing polymer concentration gave greater circularity and smaller cell size. Of particular interest was that greater scaffold stiffness correlated with increased migration velocity, indicating a focal adhesion independent mechanism for migration, like amoeboid migration, that is promoted by increasing stiffness. Porous scaffolds have been of particular interest as bone mimics due to their ability to recapitulate the mechanical and physiological conditions of the bone, alongside promoting cell differentiation and osteogenic markers in the study of mesenchymal stem cells (Ramírez-Rodríguez et al., 2017[195]; Wang et al., 2021[238]). Bassi et al. (2020[17]) developed a hydroxyapatite scaffold by a foaming process followed by a sintering step and a hybrid scaffold composed of collagen and magnesium-hydroxyapatite was formed via freeze drying of a hydrogel. Cells grown in monolayer and spheroids enriched for stemness generated in low attachment conditions were both seeded onto the scaffolds and both were found to interact with the bone-mimicking scaffolds. Spheroids seeded on the two scaffolds demonstrated upregulation of various genes linked to stemness including OCT4, NANOG, IL6 and HIF-1α when compared to scaffold-seeded monolayer cells. These findings indicate that focusing solely on cell-ECM interactions are limiting, particularly when studying features such as stemness. Incorporating models with cell-ECM and cell-cell interactions will be necessary to replicate *in vivo* work, as cancer research demands additional layers of complexity.

### 3D Bioprinting

The concept of bioprinting of tissues originally has its roots in substituting damaged tissues with a major goal of achieving organ transplantation (Langer and Vacanti, 1993[125]; Mironov et al., 2003[157]). Bioprinting started seeing considerable research in the early 2000s. Lam et al. (2002[123]) developed a rudimentary method of using natural polymers such as gelatin combined with a water-based ink binder guided via computer aided design (CAD) to yield geometrically precise scaffolds with pore networks. Wilson and Boland (2003[246]) went beyond printing polymers by modifying conventional jet-based ink printers to dispense proteins or cells in a controlled layer. This system was then developed to allow the creation of multiple layers of a solution of cells and thermosensitive gel to print a hydrogel that yields cell aggregation in 3D (Boland et al., 2003[25]). Since these findings commercial bioprinters have been developed that are commonly either extrusion based, whereby a continuous stream of a 'biological ink' (bioink) is printed onto a surface, or inkjet based, which involves the spatial placement of each individual droplet of bioink (Murphy and Atala, 2014[168]). 

These methods have shown potential in the production of various tissues by printing tissue-specific cells within a hydrogel that mimics the native ECM of the cell. Examples of these are: bone ECM mimics and chondrocytes, adipose tissue mimics alongside tissue derived MSCs and even organoid models combining pluripotent stem cells with hydrogels (Gu et al., 2020[78]). Despite the goal being to develop tissues, it has become evident that various ECM bioinks with optimizable structural and physiological properties can be developed and combined with cells to study cell-ECM interactions. However, there are unique variables to consider when producing bioinks, as they need to be compatible with the bioprinting technique. For example, in the case of laser-based bioprinting a photocurable bioink is required. Furthermore, bioinks containing cells need to maintain cell viability after the printing process (Datta et al., 2020[44]; Kim et al., 2020[106]).

Swaminathan et al. (2019[222]) looked to characterize minimal composition natural bioinks to produce hydrogel scaffold alternatives to Matrigel^TM^ using breast cancer cell lines. They identified that bioinks of alginate combined with collagen or gelatin allow spatial deposition down to the micron level using a bioprinter. Cell viability was maintained on printing in all three bioinks, however only Matrigel^TM^ supported spheroid formation from single cell deposition. To overcome this, inks containing preformed spheroids were used which gave rise to successful spheroid deposition, however irregular shapes and migration were observed after 72 hours. The printed spheroids demonstrated a greater resistance to paclitaxel than dispersed printed cultures. This further confirms the trend that the 3D organization of cells, facilitated by cell-cell contacts, has an additive effect when combined with scaffold-based systems. Whilst this study highlighted the feasibility of developing cheap and accessible bioinks, the high cost of bioprinting equipment remains a barrier in the field. To overcome this, Reid et al. (2016[197]) optimized a cost-efficient open source bioprinter with the capability to spatially print single cells. This team went on to to utilise this bioprinter with collagen bioinks and breast cancer cells to produce 3D co-cultures. Bioinks containing the non-tumorigenic MCF-12A cell line were used to create spheroids, after which tumorigenic MCF-7 cells were introduced to yield a 'chimeric' spheroid. A particularly interesting observation was that the MCF-7 cells phenotypically became less tumorigenic in this environment, as the epigenetic marker 5-hydroxymethylcytosine, which is often downregulated in cancer cells, increased to normal cell levels (Reid et al., 2019[198]). This was a curious finding as it highlights the epigenetic impact of the tumor microenvironment on the phenotype of cancer cells. 

Another team interested in the effects of bioprinting co-cultured cells in 3D to study phenotypic changes is that of Langer et al. (2019[124]). In this study, cell mixtures in a gelatin and alginate hydrogel bioink were used to print a breast cancer spheroid central core surrounded by fibroblasts. Interestingly, the proliferation marker Ki67 was less abundant in all breast cancer cell lines when compared with monolayer cultures. However, the putative invasive line MDA-MB-231 demonstrated the highest proliferation and greatest metastatic potential, reflecting known behaviour from *in vivo* research. To further study co-culture effects, MSCs were also printed into the model, whilst no effects on migration or proliferation were observed there was an increase in collagen deposition in the surrounding area. Furthermore, this team also 3D printed inks of pancreatic cancer patient derived xenografts alongside pancreatic stellate cells (PSCs) and endothelial cells. In this complex co-culture organoid, heterogeneous activation of the mTOR pathway was observed throughout the cancerous cells and the printed tissue orientated to produce structures mimicking those observed in both respective xenografts and in patients. These studies demonstrated the ability of 3D printing of singular cells, spheroids and even co-cultured organoid tissues in simple bioink scaffolds. This allowed various observations on mechanisms in tumor pathogenesis, whether in the form of drug resistance, formation of heterogeneic populations or migratory capacity in the presence of Stromal cells. Demonstrating how increasing layers of complexity can be introduced to bioinks, which cannot be replicated in monolayer cultures.

However, these models are still limited in terms of their ability to mimic ECM composition, as they use simple component bioinks or rely on the addition of stromal cells to produce ECM. Pati et al. (2014[182]) aimed to overcome this shortcoming by producing decellularized scaffolds as bioinks. This team decellularized and solubilized adipose, cartilage and heart tissues achieving 98 % cellular removal and the capability to produce this ECM scaffold as spatially desired. Whilst cancer cells were not used in this study, adipose derived stem cells and MSCs demonstrated viability after extrusion. It was shown here that a complex scaffold system could be developed of which cancer cells could be introduced to study pathogenesis. Furthermore, given that decellularization can be achieved using various tissues, this approach should allow the development of scaffolds that better recapitulate specific tumor niches. Mollica et al. (2019[162]) demonstrated this by producing decellularized rat and human mammary bioinks that could be printed alongside breast cancer cell lines. After pepsinization and dilution of the decellularized scaffold in acetic acid, a hydrogel that retained growth factor levels and maintained similar stiffness to commercially used collagen and geltrex^TM^ hydrogels was produced. Interestingly this scaffold yielded lower levels of growth factors than commonly used rat-tail collagen and higher levels than geltrex^TM^. This was reflected in variations in transcriptomic profiles of breast cancer cells on these scaffolds, as differences were seen in pathways involved in hypoxia, NF-kβ and TGF-β signaling. These findings highlight the importance of using more native scaffolds when producing 3D cultures, as scaffold composition may have a significant impact on key signaling pathways in cancer cell pathogenesis. A main goal of 3D bioprinting should be to produce bioinks from patient samples capable of yielding a scaffold reflective of the TME of patients. With such a model it would be possible to study metastatic potential and therapeutic response to develop a personalized treatment regime. However, this approach is likely limited by patient sample acquisition, accessibility of bioprinting machinery and the scalability of such a system to achieve high-throughput screening. In the meantime, simpler bioinks that are more customizable prove useful in teasing apart cell-cell and cell-ECM interactions in tumor pathogenesis.

## Advanced Techniques

Although 3D *in vitro* systems show the potential to overcome the limitations of monolayer cell culture systems, they still provide limited spatial organization and cell-cell interactions. Microfluidics emerge as an improved microscale 3D *in vitro* model for biological approaches, improving the functionality and throughput of traditional 3D culture systems. This technology manipulates small fluid volumes within microchannels with a low Reynolds number, ensuring the flow's laminar behavior is reproducible and predictable (Di Carlo, 2009[48]). Using microfluidics to create 3D cancer models enhances research at the cellular level, where molecular, chemical, and physical parameters can be precisely controlled. An essential advantage of these models is their small size and transparency, allowing for real-time microscopic observation-something impossible with animal models. Additionally, 3D microfluidic platforms can have different shapes and materials; emphasizing their tunability, they can be modified to achieve specific objectives. These devices can be made from various materials, such as poly(methyl methacrylate) (Rutz et al., 2015[204]), glass (Kim et al., 2018[110]), polycarbonate (Petta et al., 2018[187]; Rutz et al., 2015[204]), and polydimethylsiloxane (PDMS) (Alghannam et al., 2025[5]; Mazutis et al., 2013[152]). However, PDMS is commonly used to create these devices from their negative design molds due to its biocompatibility, ease of manipulation, transparency, and gas permeability (Alghannam et al., 2025[5]; McDonald and Whitesides, 2002[153]). Although oncology uses microfluidics to isolate circulating tumor cells from peripheral blood (Cai et al., 2023[28]; Chen et al., 2023[33]), their application has been extended to 3D culture. In this review, we will focus on two aspects.

### Droplet-based microfluidic system

**Figure 7:**
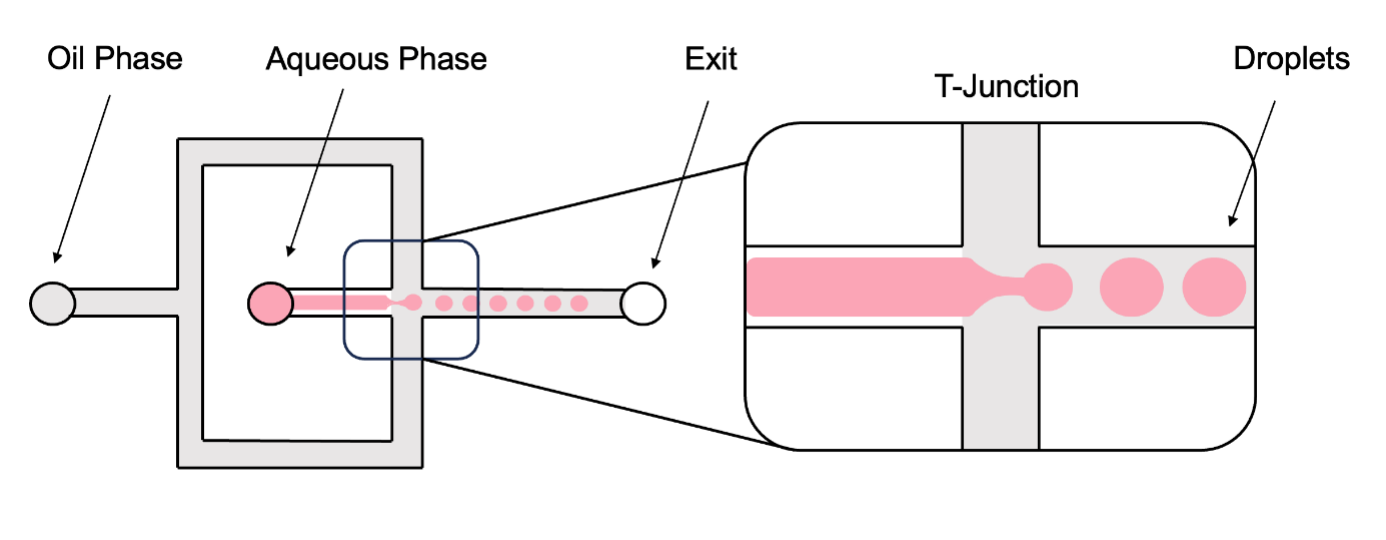
Droplet generation in a T-junction scheme. The oil phase interrupts the aqueous phase injected, due to the immiscibility of the fluids, aqueous phase droplets are generated in oil moving towards the outlet in a controlled and repeated manner as the phases are constantly injected. The artwork used in this figure was adapted from Servier Medical Art (https://smart.servier.com). Servier Medical Art by Servier is licensed under a Creative Commons Attribution 4.0 International License (https://creativecommons.org/licenses/by/4.0/).

As mentioned above, the base of 3D culture systems relies on spheroid formation. The microfluidics field offers two main advantages compared to traditional 3D systems: a smaller sample size needed to obtain the same effect and the functionality provided by spatial and temporal control, both challenging to achieve with conventional 3D systems (Sung and Beebe, 2014[220]). These advantages, in addition to the significant variability in spheroid diameter, low throughput, complex handling procedures, and static environments with fast depletion of oxygen and nutrients observed in conventional 3D culture models (Kopanska et al., 2016[114]; Štampar et al., 2020[219]), present droplet-based microfluidics as a promising tool. Droplet-based systems have recently been developed for drug screening on multicellular aggregates or single cells (Mazutis et al., 2013[152]; Sart et al., 2022[208]). The predictability at low Reynolds numbers can be applied to the formation of homogeneous droplets (Di Carlo, 2009[48]). This technique is based on emulsifying two immiscible fluids, where an oil phase meets an aqueous phase at a junction, allowing aqueous droplet generation (Figure 7(Fig. 7)) (Dangla et al., 2013[42]; Gruner et al., 2015[76]). Offering the advantage of easily tuning the droplet size by modifying the microchannel cross-section geometry and/or flow rates (Lee et al., 2020[130]; Utech et al., 2015[233]).

From this, pharmaceutical research has employed micrometer-sized alginate particles to encapsulate living cells. Encapsulation occurs when an aqueous alginate solution with chelated calcium ions (Ca2+) containing cells is emulsified in the fluorinated oil phase crosslinked with acetic acid to achieve stable gelation (Chen et al., 2016[34]; Utech et al., 2015[233]). Initially, fluorocarbon oils were proposed as the continuous phase for their high cell survival and growth compatibility due to their gas permeability, hydrophobicity, and low solubility for biological reagents of the aqueous phase (Alghannam et al., 2025[5]; Chen et al., 2011[32]). Also, as droplets are prone to coalesce, the use of surfactant in any droplet-based application to ensure stabilization is needed (Holtze et al., 2008[87]; Zdrali et al., 2017[258]). With these considerations and a small amount of manual handling, this technique quickly generates uniform-sized cell-encapsulated droplets in a high-throughput manner.

Over the years, multiple studies with different materials, cell lines and days of culture have been carried out (Table 2(Tab. 2); References in Table 2: Chen et al., 2016[34]; Fevre et al., 2023[61]; Hoesli et al., 2017[86]; Kwak et al., 2018[120]; Lee et al., 2020[130]; Ling et al., 2022[139]; Sart et al., 2017[209]; Utech et al., 2015[233]; Zhang et al., 2022[260]). Although alginate is presented as a suitable biomaterial, it limits droplet control over quantification due to the challenge of imaging free droplets. Additionally, mammalian cells cannot interact specifically with alginate (Chen et al., 2016[34]; Huebsch et al., 2010[89]; Rowley et al., 1999[203]; Tomasi et al., 2020[230]; Utech et al., 2015[233]). To address this, peptide-functionalized alginate (Arg-Gly-Asp, RGD) provides integrin bonding sites, allowing the attachment of encapsulated cells to the alginate network via ɑv integrin for the growth of adherent cells (Huebsch et al., 2010[89]; Rowley et al., 1999[203]; Utech et al., 2015[233]). Despite the improvements, the liquid droplets made from the cell culture medium appear to facilitate the process over hydrogels (Chen et al., 2016[34]), leading the 3D cell culture in droplets toward new horizons.

The shift in focus in 3D cell culture is directed towards biomaterials that allow cell interaction with minimal stress for extended periods. Kwak et al. (2018[120]) encapsulated MCF-7 cells in their culture media through fluorinated oil with a surfactant, avoiding complex polymer hydrogel systems. The MFC-7 tumor spheroid generation was achieved with high uniformity and yield (> 1000/min, 16-20 Hz) within a droplet-based microfluidic chip. Notably, reaching droplets diameter up to 250 µm after 2 weeks, the same as Utech et al. (2015[233]). For the recovery and culture of the spheroids in the long term, the droplets were disrupted with 1H,1H,2H,2H,-perfluoro-1-octanol (PFO) added in the droplet solution. After droplet destabilization, the oil phase was discarded after centrifugation and the spheroids were resuspended in a conventional cell incubation system with magnetic stirring to prevent their fusion (Kwak et al., 2018[120]; Utech et al., 2015[233]). Years later, the same team increased the droplet generation yield by 20 % and demonstrated the applicability of droplet-generated spheroids for studying nanocomposites in oncology. In their work, the encapsulation nanocomposite with U87MG brain tumor cells decreased the viability of tumor spheroids from 91 % to 55 % after near-infrared region (NIR) irradiation, being suitable agents for 3D tumor spheroid droplets undergoing photothermal therapy (Lee et al., 2020[130]). Moreover, a high number of spheroids can be generated by embedded cells inside droplets with homogeneous volumes in an automated manner. Controlling the spheroid size and the number of cells is essential through the adjustment of the droplet volume, the continuous phase (oil) flow rate, or cell concentration (Lee et al., 2020[130]; Utech et al., 2015[233]). However, the limitation researchers faced was the demulsification and centrifugation that the generated droplets required from the oil, compromising cell viability (Hoesli et al., 2017[86]; Kwak et al., 2018[120]; Utech et al., 2015[233]). Zhang et al. (2022[260]) proposed a simple one-step approach where the generated photocrosslinkable gelatin GeLMA microspheres solidify directly in the device tubing by UV exposure. Thus, the obtained hydrogel spheres were transferred to cell culture media and demulsified through the evaporation of the oil with a low boiling point (33ºC). A notable limitation highlighted in this work is the non-homogeneous cell distribution within the hydrogels. This may affect the uniformity of the generated spheroids compared to cell sedimentation through gravity allowed by low-concentration hydrogels.

**Figure 8:**
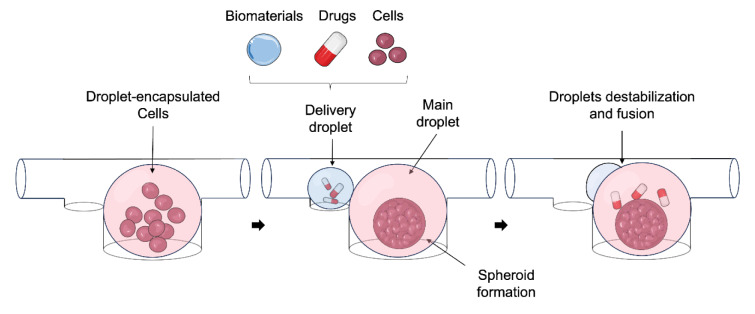
Spheroid-droplet formation and treatment/co-culture in an anchored chip workflow. Cells are encapsulated within a droplet retained in an anchored chip. Once the cells settle at the bottom of the droplet, they form the spheroid. A delivery droplet containing biomaterial, drug molecules, or cells is also created and retained in the next anchor. After droplet destabilization with 1H,1H,2H, and 2H-perfluoro-1-octanol (PFO), the fusion of the droplets is achieved, and the contents of the delivery droplet are released into the main droplet containing the spheroid. The artwork used in this figure was adapted from Servier Medical Art (https://smart.servier.com). Servier Medical Art by Servier is licensed under a Creative Commons Attribution 4.0 International License (https://creativecommons.org/licenses/by/4.0/).

Another proposal to follow up and maintain the viability of the encapsulated cells is the droplet confinement in anchors and rails (Abbyad et al., 2011[2]). Sart et al. (2017[209]) based on anchors droplet confinement, encapsulated H4-II-EC3 cells in agarose droplets through fluorinated oil, achieving 800 droplets filling 500 anchors within 3-5 minutes. This study demonstrated that after 23 hours, the encapsulated cells settled at the bottom of each droplet and formed spheroids. The viability of the formed spheroids compared with monolayer cultures remained similar above 95 % at 7 days of culture. Notably, dead cells were found near the edge of the spheroids by the agarose boundary, and no necrotic core was observed; meanwhile, in human mesenchymal stem cells (hMSC), dead cells were located in the core. This demonstrates that the device could support cell proliferation and phenotype preservation under a media-term static culture, highlighting the heterogeneity within the cell niche. Exploring the anchored microfluidic chips from Abbyad et al. (2011[2]) and Sart et al. (2017[209]), Tomasi et al. (2020[230]) performed multiple co-cultures by fusing A673 spheroids droplet with Jurkat cells droplet, H4-II-EC3 cell with E.coli and hMSC CD146bright with hMSC CD146dim. It also demonstrated the Matrigel^TM^ addition through the second droplet to the B16-F0 spheroid droplet. These devices demonstrate their ability to maintain droplets, form spheroids and track them over time (Figure 8(Fig. 8)).

The application of 3D cell culture in droplet-based microfluidics is wide and it has been recently presented. Minimal residual disease (MRD) is the microscopic clusters of treatment-resistant cells that remain after a complete clinical/radiological response and can reinitiate tumors (Luskin et al., 2018[144]). In addition to the cholesterol and lipid metabolism pathways observed in MRD (Artibani et al., 2021[10]; Velletri et al., 2022[235]), Yang et al. (2025[254]) developed an accurate experimental model to understand better MRD biology, which could be used for drug screening in solid tumors, especially ovarian cancer. OVACAR-5/RFP cells were encapsulated in biocompatible hydrogels in a surfactant-free droplet-based microfluidic platform and after obtaining the first transcriptomic characterization of clinical MRD in patients who responded to primary chemotherapy, researchers compared with the chemotherapy-resistant cells obtained from patients who were poor responders. Although both populations survive chemotherapy, they observed significant transcriptional differences. This highlights the need for distinct therapeutic approaches to target each population and the utility of 3D microtumors as a drug-testing platform.

Using an anchored chip, Fevre et al. (2023[61]) tested combinatorial drugs on Ewing sarcoma droplet spheroids. This study presented a protocol to screen the combination therapy of Cisplatin and Etoposide on spheroids over time. Interestingly, the microfluidic format does not introduce any strong bias on the measurements of IC50 over experimental periods tested compared with spheroids in a 96-well plate. They observed different drug-response dynamics between the intercalary addition of etoposide and cisplatin between days one and two, where synergistic interactions were found more effective when etoposide was applied first. This probes methods to identify fundamental mechanisms that lead to the synergy of antagonism between drugs.

On the other hand, stationary microfluidic droplet systems propose an approach to multiplexed analysis of tumor spheroid fate in the presence of an immune cell population. Ronteix et al. (2022[202]) demonstrated with an anchored chip complex model of co-culture between T cells on the B16-OVA spheroid that tumor destruction is recapitulated by two mechanisms: the collective accumulation and cooperative killing of cytotoxic T lymphocytes at the spheroid side rather than simply reflecting the sum of individual T cell activities. This possibility to track immune cell-tumor interactions with this level of complexity and detail in microfluidic systems contributes to understanding immune response heterogeneity and cooperative killing (Ronteix et al., 2022[202]; Weigelin et al., 2021[241]).

Overall, despite the promising results recapitulating the structure of the initial tumor, these responses must be treated with care due to the use of cell lines, the small volume of the droplets, which may impact viability, and the interactions between droplet by-products that may affect the responses.

### Microfluidics advanced systems: Organ-on-a-Chip

Numerous studies in static condition spheroid cultures have shown that the lower fluorescent signal intensity inside the spheroid could result from a more significant necrotic core, characteristic of aged spheroids (Kopanska et al., 2016[114]; Štampar et al., 2020[219]). More complex and dynamic systems have been proposed to address the limitations in oxygen and nutrient delivery in 3D models due to the distance to the culture medium, integrating spheroids into advanced microfluidic platforms with vascular networks. The dynamic cultures allow the renewal of the culture medium, supplying oxygen, nutrients, and shear stress generated by fluid flow that resembles conditions in venous circulation (Fan et al., 2016[57]). To achieve complex models that simulate artificial human tissue *in vitro*, organs-on-a-chip (OOAC) technology using microfluidics has been proposed. Huh et al. (2010[91]) introduced the idea of an organ-on-a-chip within a lung *in vitro* model mimicking the physiology of the basic functional unit of the human lung. These devices can integrate microchannels with tissue scaffolds. The tunable advantage of OOAC microfluidics allows the culture of diverse human cell types in smaller devices in a controlled microenvironment (Farhang Doost and Srivastava, 2024[58]). A recent study on dynamic 3D cell cultures of human blood vessel organoids within human fibroblasts and endothelial cell hydrogel demonstrated an endothelial perfusion network formed by day 7, remaining stable for over two weeks. This model achieved similar flow and shear stress rates to physiological levels, demonstrating that the endothelial layer within these models is essential for perfusion. Thus, when organoid vasculature is low, it results from increased resistance, elevated intraluminal pressure, and reduced flow, which is not only associated with shear but also with an increase in oxygen and nutrient availability (Quintard et al., 2024[193]).

In cancer research, impactful applications in the generation of tumor-on-a-chip (TOAC) models enable replicating the cellular composition and microarchitecture of native tumors by applying specific functions in a 3D format. Villasante et al. (2024[236]) proposed a microfluidic model for the alternative vasculature in neuroblastoma (NB), demonstrating the role of shear stress in promoting vascularization. Using a collagen-based biomaterial with 36 dynes/cm^²^ (143 µL/min), the established range observed in healthy vessels sustained that transdifferentiations occur within the context of pre-existing healthy capillaries from the host. They demonstrated capillary formation through the co-culture of endothelial and NB cells, which required an initial shear stress. Something completely opposite to the cell death observed in static monolayer and 3D cell culture conditions. The successful application of this shear stress in the NB-on-a-chip models demonstrates a crucial precursor for the transdifferentiation processes in neuroblastoma. In addition, it highlights the potential for simulating physiological conditions and improving the accuracy of the NB microenvironment representation. This model is a valuable tool to explore specific aspects of cancer vascularization and transdifferentiation, offering insights into the dynamic interplay between tumor cells and the endothelial microenvironment. 

These advanced microfluidic systems can be used to study key stages in cancer progression, such as intravasation and migration. Transwell assays with and without endothelial layers are used to measure cell migration *in vitro* (Muller, 2003[167]). However, this methodology lacks microenvironmental factors such as matrix or fluid flow, which have been demonstrated to influence cell migration (Mohamadian Namaqi et al., 2024[158]). For example, Natural Killer (NK) cells require a series of adhesion steps to the endothelial layer of blood or lymphatic vessels to achieve the process of trans-endothelial migration (Muller, 2003[167]). Also, NK cells exhibit different migration and contact dynamics depending on their phenotype, cell differentiation stage, and IL-2 activation (Lee and Mace, 2017[128]; Olofsson et al., 2014[174]). So, studying their interaction in a vessel-like system can deepen the understanding of the cellular interactions and immune responses under flow conditions. 

Moreover, studying drug delivery techniques with 3D vascularized microfluidics cancer model could improve the antitumor treatment. Zhao et al. (2023[263]) demonstrate enhanced cancer therapy by applying ultrasound with microbubbles to enhance the extravasation and intra-tumoral distribution. Barros et al. (2025[16]) developed a skin/skin cancer-on-a-chip microfluidic platform resembling native skin tissue by confirming the formation of differentiated epidermis and ridges at dermal-epidermal junctions. Simulating tumor invasion by embedding musculus skin melanoma cells (MCs) beneath the epidermis, doxorubicin-loaded GelMA microneedles (MNs) were tested for localized transdermal drug delivery. They confirmed the robustness of the proposed model for therapeutic application where the drug delivery through microneedles demonstrated a significantly higher efficiency than diffusion through media flow, penetrating uniformly at 600 um depth and reaching melanoma cells.

In summary, these complex models offer a remarkably adaptable platform in which a small sample in a cost-effective and time-efficient manner expedites valuable insights into cancer biology and potential treatments. Recreating the most accurate microenvironment allows us to replicate the vasculature influence in drug delivery, mechanisms of cancer cell intravasation, and extravasation. Thus, providing a clear big picture of the metastatic process and potential therapeutic targets. The complexity of tumor and microenvironmental heterogeneity sets the starting point for the complex and advanced *in vitro* culture models to be used to obtain accurate and applicable results.

## Conclusion

The complexity of cancer has motivated the interest of scientists to elucidate it. For this purpose, the first method was cell culture in monolayers, which presents a somewhat controlled set of parameters. However, it is a reductionist model compared to the complexity of the disease. From that, 3D cultures models have emerged as a promising method for oncology research. In this review we describe and compare the limitations and advantages of each technique developed over the years. As promising as it is, the choice of culture method represents a challenge where the scientific question answers the purpose of the method of choice. For functional studies, spheroids generated from the scaffold-free techniques as liquid overlay and hanging drop techniques would be a good choice. However, for the study of the microenvironment and drug resistance it depends on the type of cancer the scaffold-based method to be used. Likewise, the use of static scaffolds does not allow the inclusion of fluidic processes, such as the metastatic process. For this case, advanced techniques using microfluidics would be more appropriate. Even using more complex models such as 3D bioprinting or organ-on-a-chip would allow to reproduce the cancer in a more reliable way along with the development of a personalized medicine for each patient. 3D culture models promise to reduce the gap between *in vitro* and *in vivo* experiments by increasing their complexity; however, the proper choice of model must be with appropriate caution and planning. Ultimately, oncology research promises an important field of technological development where interdisciplinary work between biology, medicine and engineering is a key part of the development of personalized cancer medicine.

## Notes

Isidora Panez-Toro and Joshua Mountford contributed equally as first author.

Javier Muñoz-Garcia and Dominique Heymann (Nantes Université, CNRS, UMR6286, US2B, Biological Sciences and Biotechnologies unit, Nantes 44322, France; E-mail: dominique.heymann@univ-nantes.fr) contributed equally as corresponding author.

## Declaration

### Acknowledgments

JM is funded by the STRIKE project HORIZON-MSCA-2021-DN-01, grant number 101072462. IP-T is supported by the Region des Pays de la Loire (France) and the Institut of Cancérologie de l'Ouest (Saint-Herblain, France). The works was supported by a grant from the Fondation de l'Avenir referenced AP-RM-23-021.

### Conflict of interest

No conflict of interest to declare.

### Use of artificial intelligence (AI)

AI has not been used for the preparation of the main text of manuscript. Figures were created using BioRender.com (https://BioRender.com/y3tr263).

## Figures and Tables

**Table 1:**
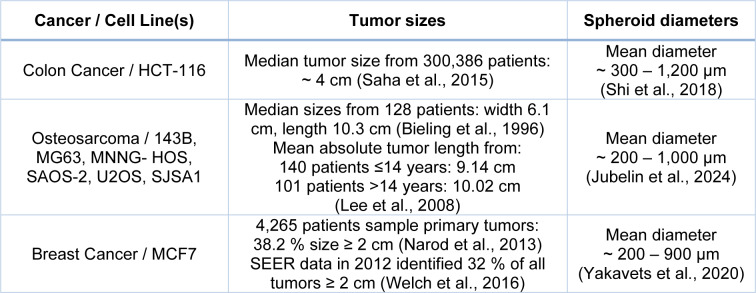
Examples of spheroid size compared to patient tumor sizes

**Table 2:**
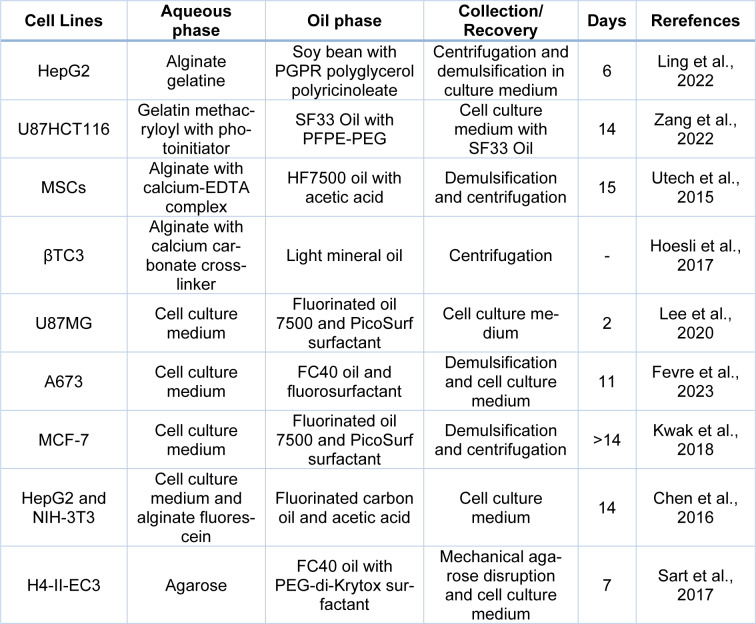
Droplet cell encapsulation methods with different materials

**Figure 1:**
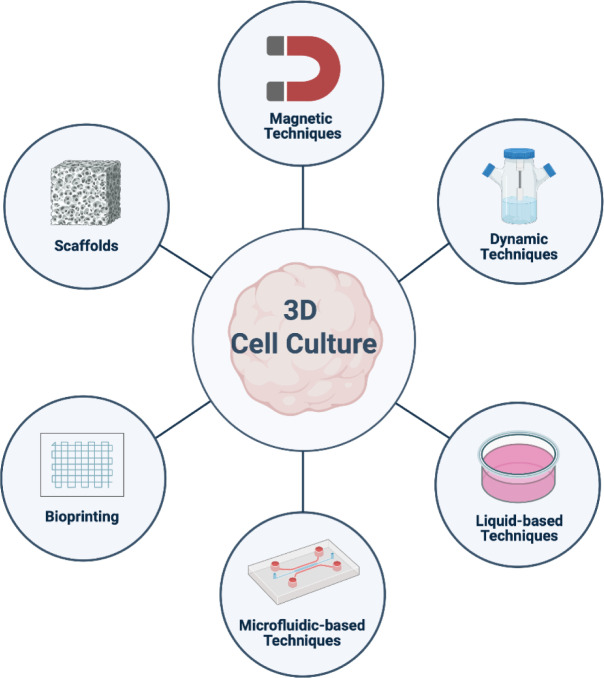
Graphical abstract
